# Clinical Characteristics of COVID-19 Patients in a Regional Population With Diabetes Mellitus: The ACCREDIT Study

**DOI:** 10.3389/fendo.2021.777130

**Published:** 2022-01-13

**Authors:** Daniel Kevin Llanera, Rebekah Wilmington, Haika Shoo, Paulo Lisboa, Ian Jarman, Stephanie Wong, Jael Nizza, Dushyant Sharma, Dhanya Kalathil, Surya Rajeev, Scott Williams, Rahul Yadav, Zubair Qureshi, Ram Prakash Narayanan, Niall Furlong, Sam Westall, Sunil Nair

**Affiliations:** ^1^ Department of Diabetes and Endocrinology, Countess of Chester Hospital NHS Foundation Trust, Chester, United Kingdom; ^2^ School of Computer Science and Mathematics, Liverpool John Moores University, Liverpool, United Kingdom; ^3^ Department of Diabetes and Endocrinology, Arrowe Park Hospital, Birkenhead, United Kingdom; ^4^ Department of Diabetes and Endocrinology, The Royal Liverpool University Hospital, Liverpool, United Kingdom; ^5^ Department of Diabetes and Endocrinology, Aintree University Hospital, Liverpool, United Kingdom; ^6^ Department of Diabetes and Endocrinology, Warrington Hospital, Warrington, United Kingdom; ^7^ Department of Diabetes and Endocrinology, Leighton Hospital, Crewe, United Kingdom; ^8^ Department of Diabetes and Endocrinology, Whiston Hospital, Prescot, United Kingdom

**Keywords:** diabetes, COVID-19, mortality, CRP, risk factors, observational study

## Abstract

**Objective:**

To identify clinical and biochemical characteristics associated with 7- & 30-day mortality and intensive care admission amongst diabetes patients admitted with COVID-19.

**Research Design and Methods:**

We conducted a cohort study collecting data from medical notes of hospitalised people with diabetes and COVID-19 in 7 hospitals within the Mersey-Cheshire region from 1 January to 30 June 2020. We also explored the impact on inpatient diabetes team resources. Univariate and multivariate logistic regression analyses were performed and optimised by splitting the dataset into a training, test, and validation sets, developing a robust predictive model for the primary outcome.

**Results:**

We analyzed data from 1004 diabetes patients (mean age 74.1 (± 12.6) years, predominantly men 60.7%). 45% belonged to the most deprived population quintile in the UK. Median BMI was 27.6 (IQR 23.9-32.4) kg/m^2^. The primary outcome (7-day mortality) occurred in 24%, increasing to 33% by day 30. Approximately one in ten patients required insulin infusion (9.8%). In univariate analyses, patients with type 2 diabetes had a higher risk of 7-day mortality [p < 0.05, OR 2.52 (1.06, 5.98)]. Patients requiring insulin infusion had a lower risk of death [p = 0.02, OR 0.5 (0.28, 0.9)]. CKD in younger patients (<70 years) had a greater risk of death [OR 2.74 (1.31-5.76)]. BMI, microvascular and macrovascular complications, HbA1c, and random non-fasting blood glucose on admission were not associated with mortality. On multivariate analysis, CRP and age remained associated with the primary outcome [OR 3.44 (2.17, 5.44)] allowing for a validated predictive model for death by day 7.

**Conclusions:**

Higher CRP and advanced age were associated with and predictive of death by day 7. However, BMI, presence of diabetes complications, and glycaemic control were not. A high proportion of these patients required insulin infusion warranting increased input from the inpatient diabetes teams.

## Introduction

As of 8 June 2021, an estimated 172 million people have been afflicted by coronavirus disease 2019 (COVID-19) with a death toll of 3.7 million worldwide (1). Severe acute respiratory syndrome coronavirus 2 (SARS-CoV-2), with ACE-2 serving as its cellular receptor, presents with a wide spectrum of clinical manifestations from asymptomatic cases to severe life-threatening pneumonia and acute respiratory distress syndrome (ARDS). Moreover, there is ongoing surveillance for variants of concern and episodic surges of cases globally (2). Therefore, the relevance of continued research about COVID-19 and its prognosis remain.

At present over 150,000 people in the United Kingdom (UK) have died with COVID-19, one of the highest case fatalities in Europe (3). According to multiple reports, risk factors for poor outcomes appear to be male sex, advanced age, diabetes, hypertension, obesity, and cardiovascular disease among others (4–6). In addition, there is evidence suggesting an increased risk of mortality among Black, Asian, and Minority Ethnic (BAME) populations (7).

The burden of diabetes on people with COVID-19 has been highlighted in several reports. In 2019, an estimated 463 million people aged between 20 to 79 years are living with diabetes worldwide and its prevalence is predicted to increase in the coming years (8). In the UK, more than 4.7 million people have diabetes (9). It is well established that certain characteristics are associated with higher mortality in patients with diabetes - in particular, cardiovascular disease (CVD), microalbuminuria, socioeconomic deprivation and foot ulceration (10, 11).

According to the ongoing International Severe Acute Respiratory and Emerging Infections Consortium (ISARIC) World Health Organization (WHO) Clinical Characterisation Protocol UK (CCP-UK) study covering 208 UK acute hospitals with over 20,000 patients, diabetes is the second most prevalent comorbidity, present in 20% of COVID-19 patients needing hospital admission. Furthermore, 26% mortality throughout the hospital inpatient population has been reported in this study (5). Therefore, identifying which clinical factors are associated with greater morbidity and mortality is of paramount importance in tackling the ongoing COVID-19 pandemic.

Two recent nationwide observational studies from France and the United Kingdom found obesity was independently associated with increased mortality and endotracheal intubation in diabetes patients with COVID-19 (12, 13). In the NHS England study, Holman and colleagues also found hyperglycaemia was associated with increased mortality. Another NHS England study found a 3-fold and 2-fold increased risk of COVID-19-related deaths amongst type 1 diabetes and type 2 diabetes respectively when compared to the general population (14). These studies provide insight into important modifiable characteristics, which can potentially improve outcomes from COVID-19 in people with diabetes. Medications commonly used in diabetes and cardiovascular disease have generated much interest especially given the integral role of ACE2 in COVID-19 pathogenesis. ACE inhibitors (ACEi) and angiotensin receptor blockers (ARB) were initially hypothesised to affect outcomes in COVID-19. However, evidence from observational studies is still largely equivocal (15–17). Whilst these studies demonstrate significant results, it remains imperative to further study regional data given the demographic variety of populations across the UK.

This study aimed to identify clinical and biochemical characteristics associated with mortality and the need for intensive care in people with diabetes and COVID-19 admitted to secondary and tertiary UK National Health (NHS) hospitals within the Cheshire and Merseyside regions of North West England.

## Research Design and Methods

### Study Design and Data Collection

This was a multicentre, retrospective, observational cohort study involving secondary and tertiary hospitals in Cheshire and Merseyside regions in the North West of England. All patients admitted to 7 hospitals in the region with COVID-19 between 01 January 2020 to 30 June 2020 with a known diagnosis either of type 1 diabetes, type 2 diabetes, other forms of diabetes, or newly diagnosed diabetes on admission were included irrespective of the reason for admission. Attribution of COVID-19 was defined as a positive SARS-CoV-2 Reverse Transcriptase PCR test. The endpoints of the study were 7 and 30 days from the date of admission and, if discharged before 7 days, general practitioners (GPs) were contacted for the occurrence of any endpoint. The study was approved by the Regional Research Ethics Committee. As ACCREDIT study has a purely non-interventional observational study design and in the context of the ongoing COVID-19 pandemic, written informed consent was not obtained from patients. The study data were collected by physicians and associates involved in the direct care of COVID-19 patients in the respective study centres from medical case notes, hospital electronic health records, local ISARIC data and the local GP practice records. Information bias due to missing data may occur. To address this, we specifically highlighted variables whose significance should be interpreted with caution.

### Clinical Outcomes

The primary outcome of the study was death by day 7 of admission. Secondary outcomes include intensive care for invasive/non-invasive ventilation, inotropic support or renal replacement within 7 days of admission and death within 30 days of admission.

We also explored diabetes-related events including insulin infusion requirement, the incidence of diabetic ketoacidosis (DKA) or ketosis, incidence of severe hypoglycaemia and the need to escalate anti-hyperglycaemic therapy (as defined by the unit increase in preadmission total daily insulin dose and/or switch from oral medication to insulin therapy).

### Statistical Analyses

Continuous variables are expressed as means (SD) and medians (IQR) and categorical variables as numbers and percentages. In determining the sample size, we used the Clopper-Pearson estimate and determined that a sample size of 700 participants with due consideration for an attrition rate of 20% for missing data and a percentage of 16% of our main outcome, would give us a 95% CI equal to 13.1% to 19.4%.

Progression of the outcome over time was characterised with Kaplan-Meier curves. To establish the robustness of the multivariate analyses, the cohort was split into separate datasets for model development and validation, with a further split into training and testing data to allow for model optimisation on out-of-sample data. Age and CRP were log-transformed before the analysis to account for skew. Multivariate logistic regression was applied using SPSS 26.0 with model selection by stepwise feedforward modelling with appropriate regularisation (Akaike and Bayesian information criteria) (18).

## Results

The ACCREDIT study included 1004 participants with diabetes and microbiologically confirmed COVID-19 admitted to 7 North West hospitals in the UK from 01 January 2020 to 30 June 2020. A total of 241 patients met the primary outcome of death within 7 days of hospital admission. The overall event rates are 24% for death by day 7 and 33% by day 30. One hospital was unable to obtain data on CRP, BMI, weight, and presence of COPD (n = 123). Upon statistical review of these data, they were found to be missing completely at random and were not included in further statistical analysis.

The clinic characteristics of this cohort are shown in **Table 1**. Mean age was 74.1 ± 12.6 years and these patients were predominantly men (60.7%). The great majority had type 2 diabetes (93.8%), followed by type 1 diabetes (4.8%), and the rest had other forms of diabetes (1.3%). The median BMI was 27.6 (25th-75th percentile 23.9 - 32.4) kg/m^2^. The mean HbA1c value was 61.2 ± 19.7 mmol/mol (7.7 ± 4%).

**Table 1:** Clinical characteristics of ACCREDIT participants according to the primary outcome of death within 7 days of admission.

Clinical features	Number of patients	Mean [± SD] or proportion (%)	Median [± IQR]	Primary outcome (*n* = 241)	Odds ratio
**Pre-admission variables**					
Age (years)	1004	74.1 [61.5, 86.7]	77 [66-84]	<0.001	
Sex	1004			>0.05	
Male		609/1004 (60.7)			
Female		395/1004 (39.3)			
Type of diabetes	1003				
Type 1		48/1003 (4.8)		0.05	0.43 [0.18, 1.01]
Type 2		941/1003 (93.8)		0.04	2.52 [1.06, 5.98]
Other		6/1003 (0.5)		–	–
Diagnosed on admission		9/1003 (0.8)		–	–
Weight (kg)	736	81.2 [79.6, 82.8]	78.5 [66.1, 92.3]	0.47	
BMI	687	28.9 [28.4, 29.4]	27.6 [23.9, 32.4]	0.52	
<18.5 kg/m^2^		16/687 (2.3)			
18.5-24.9 kg/m^2^		207/687 (30.1)			
25-29.9 kg/m^2^		205/687 (29.8)			
30 kg/m^2^ and above		259/687 (37.7)			
Ethnicity	1004			>0.05	
White		952/1004 (94.8)			
Black African, Caribbean, or Black British		11/1004 (1.1)			
Asian		9/1004 (0.9)			
Mixed or multiple ethic groups		3/1004 (0.2)			
Other ethnic group		8/1004 (0.7)			
Unknown		21/1004 (2.1)			
Index of Multiple Deprivation^a^	959			>0.05	
1		296/959 (30.9)			
2		138/959 (14.4)			
3		84/959 (8.8)			
4		91/959 (9.5)			
5		54/959 (5.6)			
6		58/959 (6.0)			
7		60/959 (6.3)			
8		58/959 (6.0)			
9		63/959 (6.6)			
10		57/959 (5.9)			
Smoking status	1004			>0.05	
Never	364	364/1004 (36.3)			
Current	67	67/1004 (6.7)			
Ex-smoker	327	327/1004 (32.6)			
Unknown	246	246/1004 (24.5)			
Duration of diabetes (in years)	696	12.4 [11.8, 13.0]	12 [6, 17]	0.61	
Latest HbA1c (mmol/mol)	943	61.2 [60.0, 62.5]	56 [47, 71]	0.05	
UACR (mg/mmol)	595	23.4 [16.8, 30.0]	2.5 [0.77, 9.8]	0.86	
Insulin		273/997 (27.4)		0.38	0.86 [0.62, 1.20]
DPP4 inhibitor		181/1001 (18.1)		0.81	0.95 [0.65, 1.40]
ACE inhibitor		278/998 (27.9)		0.21	0.81 [0.58, 1.13]
ARB		129/999 (12.9)		0.49	1.16 [0.76, 1.77]
Hypertension		599/986 (60.8)		0.15	0.80 [0.60, 1.08]
COPD		150/870 (17.2)		0.12	1.37 [0.93, 2.04]
Documented foot ulcers		95/928 (10.2)		0.08	0.6 [0.34, 1.06]
IHD		429/991 (43.3)		0.56	1.09 [0.81, 1.46]
CVD		186/998 (18.6)		0.17	1.29 [0.90, 1.84]
PVD		133/955 (13.9)		0.28	1.25 [0.83, 1.90]
CKD		302/994 (30.4)		0.02	1.45 [1.06, 1.97]
Peripheral neuropathy		144/783 (18.4)		0.98	1.01 [0.66, 1.54]
Retinopathy		221/734 (30.1)		0.34	0.83 [0.57, 1.21]
Microvascular complications^b^		498/1004 (49.6)		0.17	1.23 [0.92, 1.64]
Macrovascular complications^c^		564/1004 (56.2)		0.21	1.21 [0.90, 1.62]
**Admission variables**					
Random non-fasting blood glucose (mmol/l)	949	9.5 [9.2, 9.8]	8.2 [6.1, 11.4]	0.87	
CRP (mg/l)	858	97.2 [91.4, 103.0]	76.5 [34, 136]	<0.001	
Absolute lymphocyte count (10^9^/l)	992	1.06 [1.0, 1.15]	0.88 [0.6, 1.2]	0.001	
Troponin (ng/l)	238	168.0 [48.9, 287.1]	27.2 [13, 58.3]	0.27	
D-dimer (ng/ml)	166	1952 [1528, 2376]	1071 [524, 2120]	0.04	
Insulin infusion required		97/988 (9.8)		0.02	0.5 [0.28, 0.9]
DKA or ketosis		27/996 (2.7)		0.49	0.7 [0.26, 1.88]
Escalation of insulin or anti-hyperglycaemic therapy		117/980 (11.9)		0.22	0.74 [0.46, 1.20]
Severe hypoglycaemia		52/998 (5.2)		0.40	0.74 [0.37, 1.50]
Steroid use during admission		124/991 (12.5)		0.64	0.9[0.57, 1.40]
Oxygen use during admission		654/880 (74.3)		<0.001	7.76 [4.24, 14.21]

a Index of Multiple Deprivation presented in deciles, 1 being the most deprived and 10 being the least deprived.

b composite of CKD, retinopathy, and neuropathy.

c composite of IHD, CVD, and PVD.

UACR, urine albumin:creatinine ratio; DPP4, dipeptidyl peptidase-4; ARB, angiotensin receptor blocker; COPD, chronic obstructive pulmonary disease; IHD, ischaemic heart disease; CVD, cerebrovascular disease; PVD, peripheral vascular disease; CKD, chronic kidney disease; CRP, C-reactive protein; DKA, diabetic ketoacidosis.

The comorbidities for this group included hypertension (60.8%), ischaemic heart disease (43.3%), peripheral vascular disease (13.9%), cerebrovascular disease (18.6%), chronic kidney disease (30.4%) and COPD (17.2%). Microvascular and macrovascular complications of diabetes were evident amongst 49.6% and 56.2% of the cohort respectively. For smoking status, 6.7% were current smokers and 32.6% were ex-smokers.

For pre-admission pharmacotherapy, 27.4% were prescribed insulin therapy whilst 18.1% received dipeptidyl peptidase 4 (DPP-4) inhibitors. ACE inhibitors were prescribed in 27.9% of patients whilst 12.9% were on ARBs.

The distribution of socioeconomic status is heavily skewed with 45% amongst 959 participants belonging, respectively, to the two most deprived deciles in the UK by Index of Multiple Deprivation (19). The majority of the participants were White (96.8%).

Focusing on parameters during admission, as expected, a median CRP of 76.5 (34-136) mg/ml was observed as evidence of an ongoing inflammatory process in the majority of cases reviewed. The median absolute lymphocyte count was 0.88 (0.6-1.2) x 10^9^/l. Median random non-fasting blood glucose at the time of admission was 8.2 (6.1-11.4) mmol/l. Only 16.5% and 23.7% of the study population had available D-dimer and troponin level respectively. The median D-dimer was 1071 (524-2120) mg/ml and the median troponin was 27.2 (13-58.3) mg/l at the time of COVID-19 diagnosis. 12.5% of the cohort received corticosteroid therapy during their hospital stay.

Focusing on diabetes-related events, 9 patients were diagnosed with new diabetes during their admission with COVID-19. Escalation of anti-hyperglycaemic therapy was necessary for 117 patients (11.9%), and 97 patients required intravenous insulin infusion (9.8%). A composite of diabetic ketoacidosis and ketosis was evident in 27 patients (2.7%) and 52 patients developed severe hypoglycaemia (5.2%).

### Factors Associated With the Primary Outcome

**Figure 1:**
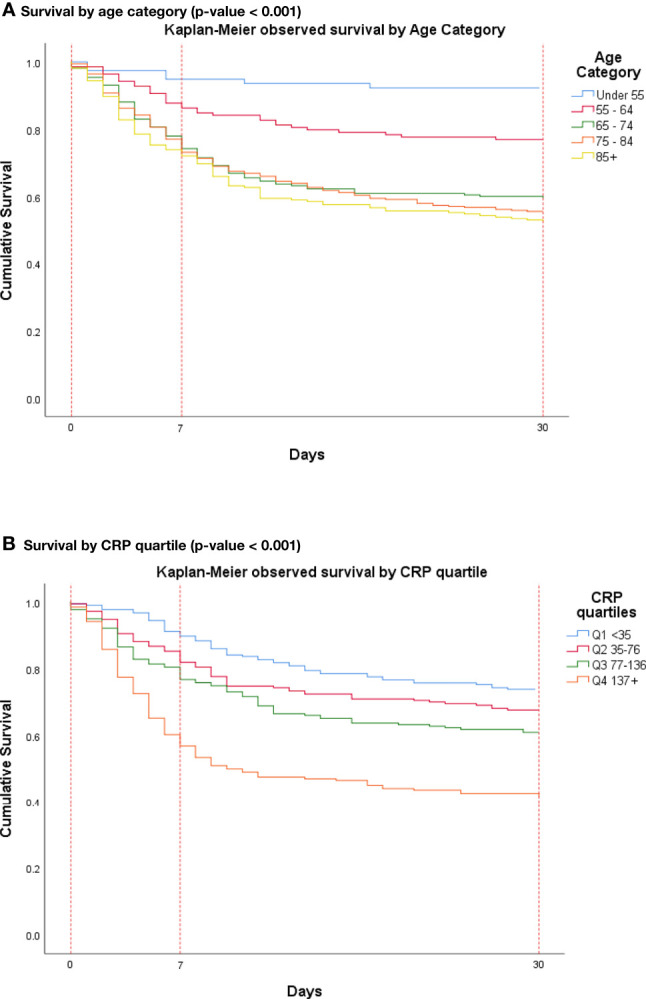
Kaplan-Meier cumulative survival probability in hospitalised patients with diabetes and COVID-19 by day 30. **(A)** Mortality was significantly higher amongst patients with advancing age. **(B)** Mortality was significantly higher amongst patients with higher CRP levels. Confidence intervals are found in the **Appendix** section (**Tables A1, A2**).

Univariate analysis revealed that amongst the pre-admission variables, age was significantly associated with the primary outcome of death by day 7 (mean age 74.1, p = <0.01). **Figure 1A** shows a clear separation for the groups stratified by age. BMI was not found to be significantly associated with mortality even after stratification based on age and WHO classification. Univariate logistic regression with intensive care admission as the outcome variable showed statistical significance for BMI (p-value = 0.007) when used alone in the model. However, when the model was adjusted for age and sex, BMI was no longer significant (p-value = 0.168). Most recently recorded HbA1c, presence of CKD, and a diagnosis of either type 1 diabetes or type 2 diabetes were modestly associated with the primary outcome (p<0.05). Moreover, when stratified by age, younger patients with CKD (age < 70 years) showed an increased risk of the primary outcome [OR 2.74 (1.31-5.76)]. Duration of diabetes, UACR, presence of macro- and microvascular complications, and use of insulin, DPP4 inhibitor, ACE inhibitor, and ARB were not associated with the primary outcome. Within our review, a series of Kaplan-Meier charts were performed for key variables identified in the univariate analysis. In this exploratory analysis, the Kaplan-Meier charts were for death up to 30 days.

Amongst the individual admission variables, only CRP was significantly associated with the primary outcome (p = <0.001). For the Kaplan-Meier curve (**Figure 1B**), CRP values were stratified into quartiles. There is an association between a greater CRP numeric value (top quartile >136 mg/L), and mortality at 30 days. This key association between CRP and mortality at seven days follows the same trajectory for up to 30 days (see **Figure 1B**). The use of supplemental oxygen was also associated with the primary outcome (p < 0.001, OR 7.76 [4.24, 14.21]. D-dimer, absolute lymphocyte count, oxygen requirement, and insulin infusion requirement were found to be significant albeit to a lesser degree (p<0.05). The use of corticosteroid therapy during admission was not associated with mortality.

### Multivariate Analysis

We determined sample sizes of 564, 245 and 195 for the training, testing, and validation datasets, respectively, with the incidence listed in **Table A3** (see **Appendix**) and without any rows with missing values gives prevalence [CIs] for the event rates of 25.25% [21.94%, 29.10%], 23.23% [18.46%, 28.80%] and 20% [14.99%, 26.17%]. The validation data set contains exclusively cases from different hospitals from those contributing to the training and test data sets.

Among pre-admission variables, the multivariate study identified age to be significantly associated with death by day 7 (p-value < 0.05). Further multivariate analysis including admission variables found that CRP was the most significant independent effect for death by day 7, followed by age. All models were fitted to the training dataset, identifying the most significant variables using AIC and BIC applied to both the training and test datasets. Performance statistics were calculated for all three data sets.

**Figure 2:**
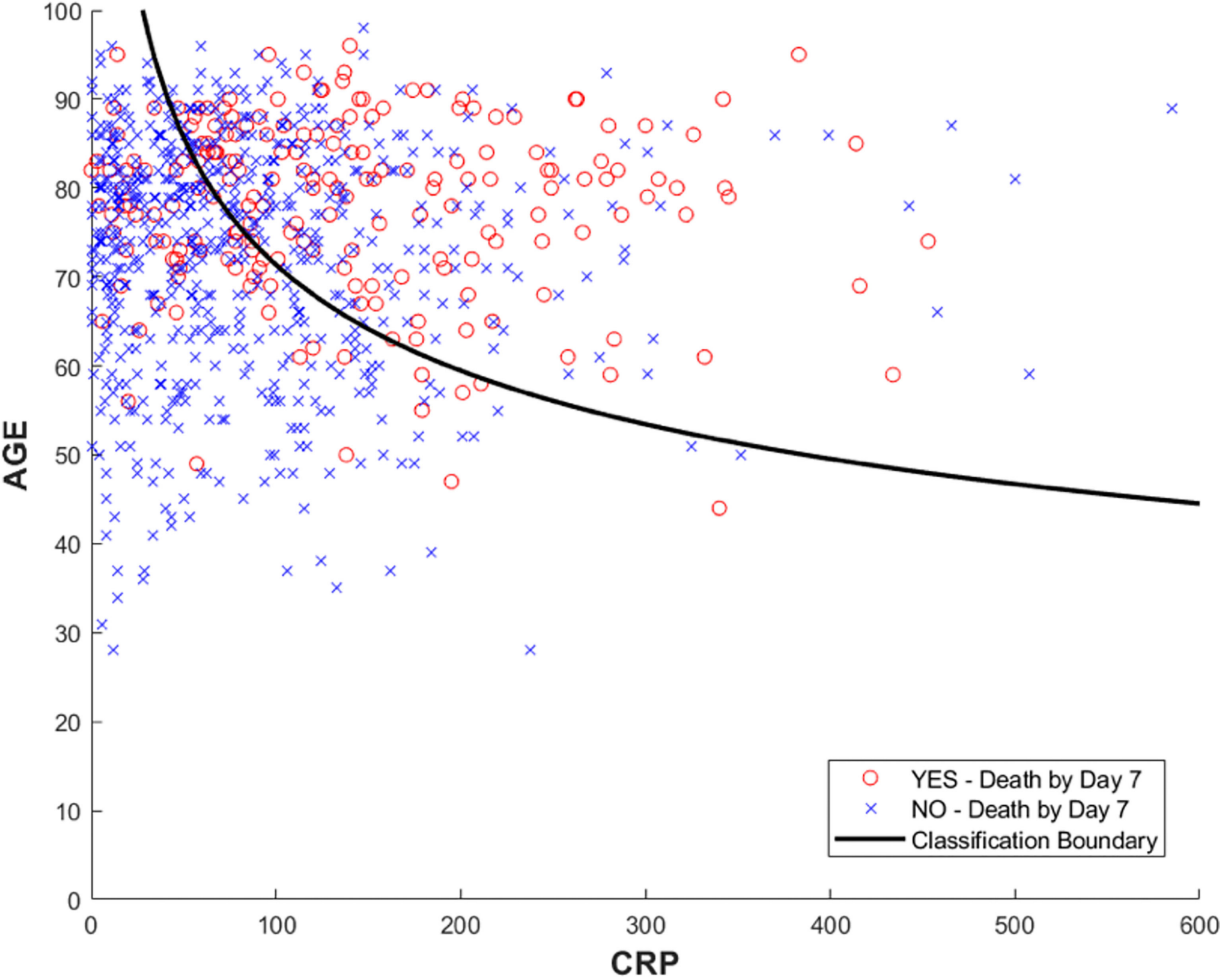
The figure shows the individual values in the training data for Age and CRP colour coded for outcome, with observed death by day 7 from admission highlighted in red. The curved line shows where the logistic regression model prediction is equal to the prevalence of events on the training data. The curved boundary results from the use of logarithmically transformed variables in the original logistic regression model. Stratifying the observations to the right of the curve and those to the left into high- and low-risk cohorts results in an odds ratio of 3.44 [2.17, 5.44] for the increase in probability of death by day 7.

We can verify the discriminant boundary for the two variables selected by direct visualisation in **Figure 2**.

## Discussion

The ACCREDIT study aimed to identify characteristics associated with 7-day mortality and intensive care admission in people with diabetes and COVID-19. Our study found CRP to be the most significant independent variable associated with 7-day mortality more so than age, and we have assessed that both parameters combined provide reasonable predictive power for this outcome. This is in keeping with other whole observational studies and metanalyses, which have identified that advanced age and CRP predisposes COVID-19 patients to a higher risk of ventilator support and death in people with or without diabetes (12, 13, 20, 21). CRP has previously been found to be positively correlated with the diameter of lung lesion and severe presentation of COVID-19 (22).

The 7-day mortality rate amongst our cohort (24%) was beyond double the rate found in the CORONADO study, which was 10.3% on day 7 of admission (12). One notable difference between the cohorts was the mean age of the CORONADO study cohort was 69.8 compared to 74.1 for the ACCREDIT study cohort (12). We also note significant socioeconomic deprivation within our cohort. Both of these factors may partially explain the significantly increased mortality revealed in the ACCREDIT population. Furthermore, data specific for persons with diabetes extrapolated from the ISARIC study revealed a 14-day in-hospital mortality rate over 29% in comparison to an estimated 26% in persons without diabetes (5). Although this finding is not directly comparable to our outcomes, it remains consistent with the mortality trend over time of this cohort.

A particular area of interest was the effects of glycaemic control on COVID-19 outcomes. Findings from previous studies have shown an association between poorer long-term and inpatient glycaemic control and greater mortality (4, 13, 23). However, in this study, both HbA1c and random blood glucose on admission were not associated with the primary outcome. Interestingly, almost a quarter in our cohort had HbA1c recorded below 48 mmol/ml, which may have contributed to the lack of association. It is noteworthy that this study did not have a strict preceding time limit on HbA1c values to be included as compared to the CORONADO group who allowed data for HbA1c to be recruited in the six months before admission only. Even then, the CORONADO study did not demonstrate an independent association between long-term glucose control and mortality at 7 days (12). This study also found an increased risk of death amongst younger patients with diabetes complicated by CKD. A population-based study shows higher probabilities of death and intubation from COVID-19 amongst patients with diabetic kidney disease (DKD) compared to other causes of CKD (24). These findings were attributed to a chronic pro-inflammatory state and immune dysregulation. Moreover, renal tubular ACE2 receptors were found to be overexpressed in DKD as compared to the general population, possibly enhancing viral entry (25, 26). There are concerns regarding the use of corticosteroid therapy in the setting of COVID-19 and diabetes given its potential to worsen glycaemic control. The RECOVERY trial showed the use of dexamethasone in moderate to severe COVID-19 improved clinical outcomes (27). Our study did not find any association between steroid use and 7-day mortality [p=0.64, OR 0.9 (0.57,1.40)]. However, only a small proportion of the cohort (12.5%) received steroids. This is despite the majority of the patients (74.3%) needed oxygen therapy. It is important to note that our data were gathered during the time when steroids use in COVID-19 was still largely under investigation. It was only later during the data collection period when dexamethasone was recommended as a national guideline. Larger and more focused studies are recommended to further elucidate its effect on diabetes and COVID-19 outcomes. Newly diagnosed diabetes amongst COVID-19 patients conferred a higher risk of adverse outcomes, according to some studies (28, 29). However, the very small number of this subgroup in this cohort makes it difficult to draw any significant conclusion from it.

Due to the limited numbers admitted to intensive care (7.5%) we did not proceed with further statistical analysis for this outcome because any significant result will likely be underpowered. Advanced care planning continues to play an important role in tackling COVID-19 and decisions surrounding escalation should be appropriately made within the wider clinical context including assessment of co-morbidities, performance status and resource allocation.

The ACCREDIT study population had higher deprivation and obesity levels in comparison to the rest of the UK population, which makes the results prone to selection bias. With regards to socioeconomic deprivation, the NHSE study found that there were substantially more deaths in the most deprived quintile - especially for those with type 1 and type 2 diabetes, and to a lesser extent for those without diabetes (13). However, our study did not show an increased risk of death with higher deprivation. It is worth noting that almost half of the ACCREDIT cohort belonged to the two most deprived deciles, which is higher compared to the regional population (45% versus 34.75%) (19). This may explain the absence of such association. Obesity levels were noted to be higher in comparison to the general UK population, especially amongst the under-55 age group (30). Throughout this pandemic, obesity has consistently been highlighted as a risk factor for worse outcomes with COVID-19 (2, 6). From the CORONADO study, the population median BMI was 28.4 kg/m^2^ and they found a positive and independent association with tracheal intubation and/or death within 7 days. However, this outcome was largely driven by tracheal intubation (12). Similarly, obesity (BMI ≥ 30 kg/m^2^) had a higher HR for death in the NHS England study (13). The median BMI for our patient cohort was lower at 27.6 kg/m^2^ and did not replicate the findings from both the above studies. Moreover, Holman and colleagues identified a higher HR for death in patients with low BMI (< 20 kg/m^2^) (13). We did identify a trend, albeit not significant, towards low BMI and 30-day mortality.

One strength of our study is the data collection strategy, which involved direct perusal of patient notes by clinicians thereby assuring the accuracy of recording clinically relevant data. However, there were limitations; it should be stressed that the ACCREDIT data will reflect the demographics of the local population attending hospitals throughout the region during these six months. As mentioned before, this cohort had higher levels of deprivation. Moreover, there were a higher proportion of people of White ethnicity. This makes it underpowered for further analysis focusing on the BAME demographic group. It is also worth noting that increased D-dimer levels appeared to show a trend towards increased 30-day mortality. However, there was a significant number of missing values D-dimer within the dataset leading to insufficient statistical power. This may be because we were not consistently looking for venous thromboembolic events during admission in the first wave of COVID-19. Regarding glycaemic control, there was no time limit defined in determining the latest HbA1c level to be included in this study, which may have contributed to the relatively low mean HbA1c level in this cohort. Also, whilst we were able to accrue data from admission to include glucose and CRP measurements, we did not collect serial measurements throughout the admission and we are unable to assess the trend of these results and their relationship to relevant outcomes. Lastly, this study did not include other commonly used medications amongst people with diabetes and other cardiovascular conditions including metformin, sodium-glucose co-transporter-2 inhibitors, and statins.

Our study highlights substantial resources are required in tackling COVID-19 for this patient cohort with diabetes. Nearly 10% patients needed intravenous insulin infusion. This is higher compared to the recent 2017 National Diabetes Inpatient Audit in 2017, wherein there was a steady decline of the use of insulin infusions from 11% in 2011 to 8.2% in 2017 (31). Of note, the rate of severe hypoglycaemia in this study (5%) was lower compared to 7% seen in the above audit. The significance of which remains to be a question. These measures represent increased demand on the inpatient diabetes services across the region including increased numbers of giving sets for intravenous infusions, greater inpatient diabetes nursing provision and education within the hospital setting.

Amongst patients with diabetes presenting with COVID-19 in the hospital setting, CRP and age are useful markers associated with an increased risk of death within the first week of admission, specifically amongst the most deprived regions in the community. Since these variables are easily identified early on during hospital admission, they can aid in determining treatment escalation plans and risk stratification models thereby potentially improving patient outcomes. Further work will allow clinician-researchers to establish a more robust risk stratification model on admission and perhaps a lower threshold for intensification of treatment for this patient cohort. This information may also aid in further prognostic studies looking into new variants and subsequent surges of COVID-19. Precedence has been previously set with the introduction of the ISARIC 4C Mortality Score (32). The ACCREDIT study results may aid in further development of a similar yet specific prognostic tool for diabetes patients with COVID-19 infection. Additional research is needed to elucidate any relationship between glycaemia and the prognosis of diabetes patients admitted to hospital with COVID-19. We suggest that future mechanistic studies could be considered to further explore the increased mortality for the diabetes cohort as compared to the non-diabetes population.

## Data Availability Statement

The raw data supporting the conclusions of this article will be made available by the authors, without undue reservation.

## Ethics Statement

The studies involving human participants were reviewed and approved by North East - York Research Ethics Committee NHSBT Newcastle Blood Donor Centre Holland Drive Newcastle upon Tyne NE2 4NQ Telephone: 0207 1048091. Written informed consent for participation was not required for this study in accordance with the national legislation and the institutional requirements.

## Author Contributions

DL and RW contributed to the study equally and should be regarded as co-first authors. DL, RW, HS, and SN conceived the study and conducted literature search. DL and RW developed the database for data collection and collation. All authors contributed to writing the study protocol. PL and IJ conducted the statistical analysis and contributed the figures. SN, HS, DL, and RW led the collection of data. At their respective study sites, the following authors conducted data collection from the patients’ medical notes: StW, JN, DS, DK, SR, ScW, RN, NF, SaW, RY, and ZQ. Lastly, all authors collaborated in interpreting the results. DL, RW, HS, and SN drafted the manuscript and all authors contributed to critical revisions in the manuscript. All authors contributed to the article and approved the submitted version.

## Conflict of Interest

The authors declare that the research was conducted in the absence of any commercial or financial relationships that could be construed as a potential conflict of interest.

## Publisher’s Note

All claims expressed in this article are solely those of the authors and do not necessarily represent those of their affiliated organizations, or those of the publisher, the editors and the reviewers. Any product that may be evaluated in this article, or claim that may be made by its manufacturer, is not guaranteed or endorsed by the publisher.

## References

[1] WHO. COVID-19 Weekly Epidemiological Update 43. Geneva, Switzerland: World Health Organization. (2021) p. 1–3. Available at: https://www.who.int/publications/m/item/weekly-epidemiological-update-on-covid-19—8-june-2021.

[2] Apicella M Campopiano MC Mantuano M Mazoni L Coppelli A Del Prato S. COVID-19 in People With Diabetes: Understanding the Reasons for Worse Outcomes. Lancet Diabetes Endocrinol (2020) 8(9):782–92. doi: 10.1016/S2213-8587(20)30238-2 PMC7367664 32687793

[3] UK Government. Deaths | Coronavirus in the UK. Gov.uk. (2021). Available at: https://coronavirus.data.gov.uk/details/deaths (Accessed cited 2021 Jun 14).

[4] Williamson EJ Walker AJ Bhaskaran K Bacon S Bates C Morton CE. Factors Associated With COVID-19-Related Death Using OpenSAFELY. Nature (2020) 584(7821):430–6. doi: 10.1038/s41586-020-2521-4 PMC7611074 32640463

[5] Docherty AB Harrison EM Green CA Hardwick HE Pius R Norman L. Features of 20 133 UK Patients in Hospital With Covid-19 Using the ISARIC WHO Clinical Characterisation Protocol: Prospective Observational Cohort Study. BMJ (2020) 369. doi: 10.1136/bmj.m1985 PMC7243036 32444460

[6] Zhou Y Chi J Lv W Wang Y. Obesity and Diabetes as High-Risk Factors for Severe Coronavirus Disease 2019 (Covid-19). Diabetes Metab Res Rev (2020) 37:e3377. doi: 10.1002/dmrr.3377 32588943 PMC7361201

[7] Public Health England. Disparities in the Risk and Outcomes of COVID-19. PHE Publications (2020). Available at: https://www.gov.uk/government/publications/covid-19-review-of-disparities-in-risks-and-outcomes (Accessed cited 2021 Feb 8).

[8] International Diabetes Federation. IDF Diabetes Atlas Ninth Edition 2019 Vol. 1. Brussels, Belgium: International Diabetes Federation (2019). Available at: http://www.idf.org/about-diabetes/facts-figures.

[9] Diabetes UK. Diabetes UK Facts and Statistics 2016. London, England: Diabetes UK (2016). p. 1–48. Available at: https://www.diabetes.org.uk/Documents/Positionstatements/DiabetesUKFactsandStats_Dec2015.pdf.

[10] Saluja S Anderson SG Hambleton I Shoo H Livingston M Jude EB. Foot Ulceration and Its Association With Mortality in Diabetes Mellitus: A Meta-Analysis. Diabetes Med (2020) 37(2):211–8. doi: 10.1111/dme.14151 31613404

[11] Anderson SG Shoo H Saluja S Anderson CD Khan A Livingston M. Social Deprivation Modifies the Association Between Incident Foot Ulceration and Mortality in Type 1 and Type 2 Diabetes: A Longitudinal Study of a Primary-Care Cohort. Diabetologia (2018) 61(4):959–67. doi: 10.1007/s00125-017-4522-x PMC6448990 29264632

[12] Cariou B Hadjadj S Wargny M Pichelin M Al-Salameh A Allix I. Phenotypic Characteristics and Prognosis of Inpatients With COVID-19 and Diabetes: The CORONADO Study. Diabetologia (2020) 63(8):1500–15. doi: 10.1007/s00125-020-05180-x PMC7256180 32472191

[13] Holman N Knighton P Kar P O’Keefe J Curley M Weaver A. Risk Factors for COVID-19-Related Mortality in People With Type 1 and Type 2 Diabetes in England: A Population-Based Cohort Study. Lancet Diabetes Endocrinol (2020) 8(10):823–33. doi: 10.1016/S2213-8587(20)30271-0 PMC7426091 32798471

[14] Barron E Bakhai C Kar P Weaver A Bradley D Ismail H. Associations of Type 1 and Type 2 Diabetes With COVID-19-Related Mortality in England: A Whole-Population Study. Lancet Diabetes Endocrinol (2020) 8(10):813–22. doi: 10.1016/S2213-8587(20)30272-2 PMC7426088 32798472

[15] Raisi-Estabragh Z McCracken C Bethell MS Cooper J Cooper C Caulfield MJ. Greater Risk of Severe COVID-19 in Black, Asian and Minority Ethnic Populations Is Not Explained by Cardiometabolic, Socioeconomic or Behavioural Factors, or by 25(OH)-Vitamin D Status: Study of 1326 Cases From the UK Biobank. J Public Heal (United Kingdom) (2020) 42(3):451–60. doi: 10.1093/pubmed/fdaa095 PMC7449237 32556213

[16] Bean DM Kraljevic Z Searle T Bendayan R Kevin O Pickles A. Angiotensin-Converting Enzyme Inhibitors and Angiotensin II Receptor Blockers Are Not Associated With Severe COVID-19 Infection in a Multi-Site UK Acute Hospital Trust. Eur J Heart Fail (2020) 22(6):967–74. doi: 10.1002/ejhf.1924 PMC7301045 32485082

[17] Pirola CJ Sookoian S. Estimation of Renin-Angiotensin-Aldosterone-System (RAAS)-Inhibitor Effect on COVID-19 Outcome: A Meta-Analysis. J Infect (2020) 81(2):276–81. doi: 10.1016/j.jinf.2020.05.052 PMC7255761 32474043

[18] IBM Corp. Released 2019. IBM SPSS Statistics for Windows, Version 26.0. Armonk, NY: IBM Corp (2011).

[19] Ministry of Housing Communities and Local Government. The English Indices of Deprivation 2019: Research Report (2019). Available at: https://www.gov.uk/government/publications/english-indices-of-deprivation-2019-technical-report%0A; https://assets.publishing.service.gov.uk/government/uploads (Accessed cited 2021 Feb 8).

[20] Qiu P Zhou Y Wang F Wang H Zhang M Pan X. Clinical Characteristics, Laboratory Outcome Characteristics, Comorbidities, and Complications of Related COVID-19 Deceased: A Systematic Review and Meta-Analysis. Aging Clin Exp Res (2020) 32(9):1869–78. doi: 10.1007/s40520-020-01664-3 PMC7391922 32734576

[21] Petrilli CM Jones SA Yang J Rajagopalan H O’Donnell L Chernyak Y. Factors Associated With Hospital Admission and Critical Illness Among 5279 People With Coronavirus Disease 2019 in New York City: Prospective Cohort Study. BMJ (2021) 369:m1966. doi: 10.1136/bmj.m1966 PMC7243801 32444366

[22] Wang L. C-Reactive Protein Levels in the Early Stage of COVID-19. Med Mal Infect (2020) 50(4):332–4. doi: 10.1016/j.medmal.2020.03.007 PMC7146693 32243911

[23] Bode B Garrett V Messler J McFarland R Crowe J Booth R. Glycemic Characteristics and Clinical Outcomes of COVID-19 Patients Hospitalized in the United States. J Diabetes Sci Technol (2020) 14(4):813–21. doi: 10.1177/1932296820924469 PMC7673150 32389027

[24] Leon-Abarca JA Memon RS Rehan B Iftikhar M Chatterjee A. The Impact of COVID-19 in Diabetic Kidney Disease and Chronic Kidney Disease: A Population-Based Study. Acta BioMed (2020) 91(4):e2020161–e2020161. doi: 10.1101/2020.09.12.20193235 33525210 PMC7927495

[25] Gilbert RE Caldwell L Misra PS Chan K Burns KD Wrana JL. Overexpression of the Severe Acute Respiratory Syndrome Coronavirus-2 Receptor, Angiotensin-Converting Enzyme 2, in Diabetic Kidney Disease: Implications for Kidney Injury in Novel Coronavirus Disease 2019. Can J Diabetes (2021) 45(2):162–6.e1. doi: 10.1016/j.jcjd.2020.07.003 PMC7368650 32917504

[26] Menon R Otto EA Sealfon R Nair V Wong AK Theesfeld CL. SARS-CoV-2 Receptor Networks in Diabetic and COVID-19-Associated Kidney Disease. Kidney Int (2020) 98(6):1502–18. doi: 10.1016/j.kint.2020.09.015 PMC7543950 33038424

[27] RECOVERY Collaborative Group. Dexamethasone in Hospitalized Patients With Covid-19. N Engl J Med (2021) 384(8):693–704. doi: 10.1056/NEJMoa2021436 32678530 PMC7383595

[28] Farag AA Hassanin HM Soliman HH Sallam A Sediq AM Abd Elbaser ES. Newly Diagnosed Diabetes in Patients With COVID-19: Different Types and Short-Term Outcomes. Trop Med Infect Dis (2021) 6(3):142. doi: 10.3390/tropicalmed6030142 34449740 PMC8396224

[29] Li H Tian S Chen T Cui Z Shi N Zhong X. Newly Diagnosed Diabetes Is Associated With a Higher Risk of Mortality Than Known Diabetes in Hospitalized Patients With COVID-19. Diabetes Obes Metab (2020) 22(10):1897–906. doi: 10.1111/dom.14099 PMC7283710 32469464

[30] Conolly A Craig S. Health Survey for England 2018: Overweight and Obesity in Adults and Children. London, UK: NatCen Social Resrearch, Health and Social Care Information Centre (2019). Available at: https://files.digital.nhs.uk/52/FD7E18/HSE18-Adult-Child-Obesity-rep.pdf (Accessed cited 2021 Feb 8).

[31] Rayman G National Health Service. National Diabetes Inpatient Audit 2017. (2017). Available at: https://files.digital.nhs.uk/pdf/s/7/nadia-17-rep.pdf.

[32] Knight SR Ho A Pius R Buchan I Carson G Drake TM. Risk Stratification of Patients Admitted to Hospital With Covid-19 Using the ISARIC WHO Clinical Characterisation Protocol: Development and Validation of the 4C Mortality Score. Baillie JK, Semple MG, Openshaw PJM, Carson G, Alex B, Bach B, Et Al., Editors. BMJ (2020) 370. doi: 10.1136/bmj.m3339 PMC7116472 32907855

