# Supplementary material for: Clinical Characteristics of COVID-19 Patients in a Regional Population With Diabetes Mellitus: The ACCREDIT Study
Source: Front Endocrinol (Lausanne). 2022 Jan 13;12:777130. doi: 10.3389/fendo.2021.777130 (PMC8793829; doi:10.3389/fendo.2021.777130)
Supplement: Supplementary file 1 [file DataSheet_1.zip › Appendix.DOCX]

**Clinical characteristics of COVID-19 patients in a regional population with diabetes mellitus: the ACCREDIT study**

**Supplementary Material**

**Appendix**


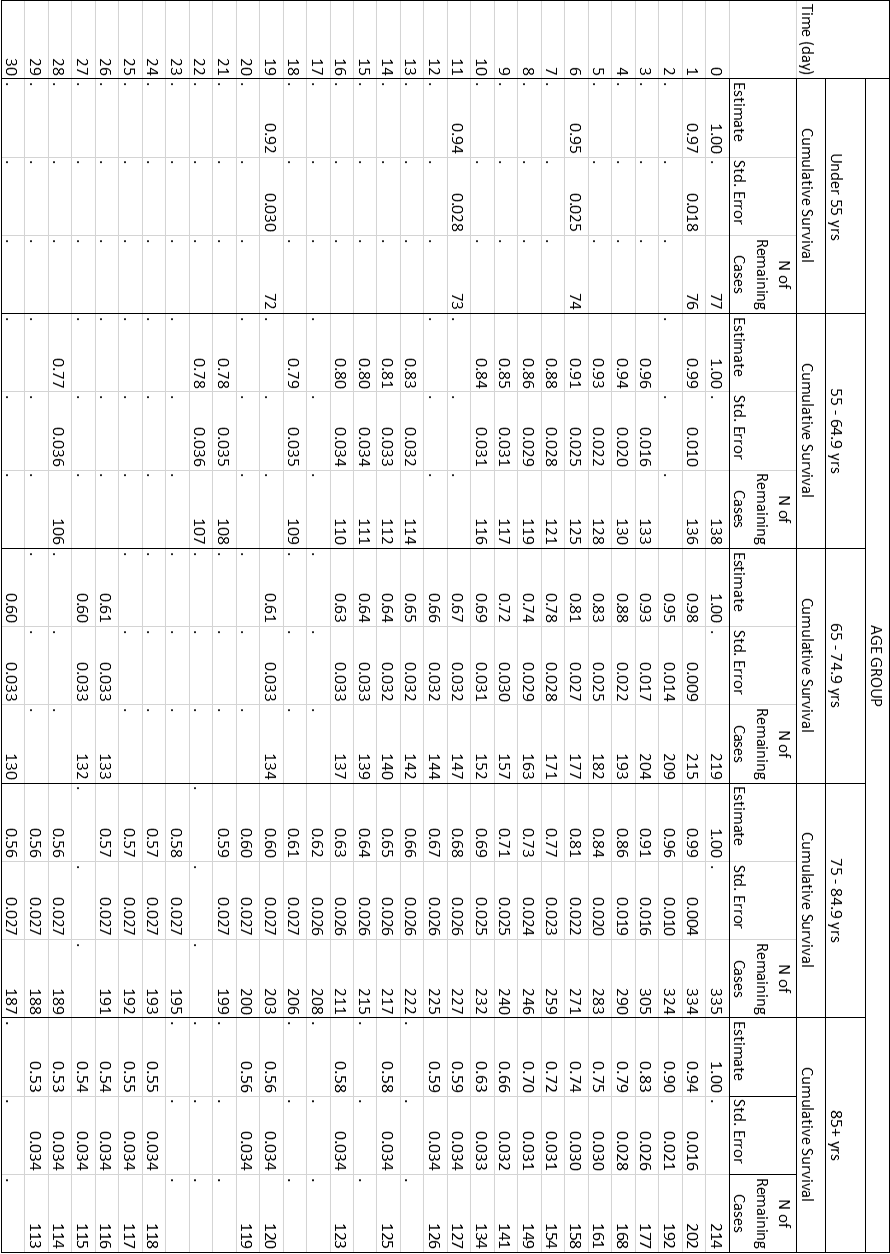


Table A1. Cumulative Survival details in relation to the Kaplan-Meier curve for age and the primary outcome in Figure 1A.

Table A2. Cumulative Survival details in relation to the Kaplan-Meier curve for CRP Groups and the primary outcome in Figure 1B.

| Hospital Index | Training data | Deaths in the training data | Testing data | Deaths in the testing data | Validation data | Deaths in the validation data | Total data | Total  events |
| --- | --- | --- | --- | --- | --- | --- | --- | --- |
| 1 | 128 | 23 | 63 | 14 |  |  | 191 | 37 |
| 2 | 124 | 37 | 61 | 11 |  |  | 185 | 48 |
| 3 | 92 | 25 | 36 | 9 |  |  | 128 | 34 |
| 4 | 106 | 31 | 40 | 9 |  |  | 146 | 40 |
| 5 | 114 | 27 | 45 | 16 |  |  | 159 | 43 |
| 6 |  |  |  |  | 83 | 10 | 83 | 10 |
| 7 |  |  |  |  | 112 | 29 | 112 | 29 |
| Total | 564 | 143 | 245 | 59 | 195 | 39 | 1004 | 241 |

Table A3. Train and test data involving CRP and age in relation to death by day 7 was performed by assigning hospital number 1 to 5 into a training set (70%) and test set (30%) respectively. We then applied the same model to hospital 6 and 7 as the validation set to ensure results were replicated.
